# A non-destructive technique using digital holographic vibrometry and Lamb waves for quality determination of polymer-metal laminates

**DOI:** 10.1038/s41598-022-22853-2

**Published:** 2022-10-27

**Authors:** Jagoda Nowak-Grzebyta, Ewa Stachowska, Frans Meijer, Tomasz Sterzyǹski

**Affiliations:** Faculty of Mechanical Engineering, Poznan University of Technology, ul. Piotrowo 3, Poznan, 60-965 Poland

**Keywords:** Optical techniques, Materials science, Optics and photonics

## Abstract

We used digital holographic vibrometry (DHV) as a non-destructive method to detect debonding areas in laminates made of aluminum and polymer (polylactide, polyvinylidene fluoride or polycarbonate). At low frequencies (up to 30 kHz) $A_0$ Lamb waves were excited and the amplitude and the phase patterns of the vibration of the sample were simultaneously registered for metal and polymer side of the laminate. Based on these patterns debonding areas in laminates were localized. The transmission properties at low frequencies were also studied in terms of: the frequency range for which regular Lamb waves have been observed, Lamb wave amplitudes and Lamb wave propagation velocity depending on the frequency. We have shown that these properties also change when a defect occures in the laminate. Even when we could not localize the defect it was still possible to detect if a sample was damaged based on the behaviour of the Lamb waves.

## Introduction

The use of polymer-metal laminates in recent years has been gaining popularity. This is due to the fact that they are lighter and have better physical properties such as elasticity modulus, tensile and flexural strength, toughness, etc. compared to pure metal or polymer components^1^. They can additionally be easily adapted to specific applications and needs.

These laminates are used in various industries, including aviation, automotive, medical equipment, household appliances and others. More and more companies in the automotive industry are currently trying to reduce the weight of vehicles in order to improve their operating parameters and reduce production costs. The same tendency can be observed in the aviation and the shipbuilding industry. Metal and polymer, when created properly, can form an element that can be easily mounted in mechanical structures.

The choice of materials for both partners of the laminate should be based on properties such as limited differences in thermal expansion, compliance with environmental conditions, electrical and thermal conductivity, the ability to dampen structural vibrations and others.

Assembling of both materials is nowadays achieved directly during processing without any additional steps, using common procedures such as injection moulding with metal inserts^2–7^. Such joining methods are particularly attractive due to the possibility of easy and complete recycling of polymer and metal components. Short-timed production cycles of the metal-polymer joints may however lead to connection defects.

Despite the growing familiarity with the production methods of polymer-metal laminates, the susceptibility to the formation of hidden or barely visible defects is still a major concern. Such defects can appear both, during the production and exploitation of a given element and can remain hidden by standard inspections^1^. Failures of this type can progressively increase over the course of operation if left undetected and ultimately have catastrophic consequences for the entire structure^1^.

While surface defects in such materials can be quite easily localized, internal (hidden) defects, the presence of which may have a significant impact on the strength of the final product, are difficult to detect. Even if detected, its localization is also necessary in order to remove or replace only the defective panel or part of the structure.

Non-destructive testing (NDT) is a useful tool for checking individual components for possible hidden defects. More than a few NDT techniques are nowadays in use, such as ultrasonic testing^8–10^ (including ultrasonic vibrometry^11^), radiography^12–14^, scanning laser Doppler vibrometry^15,16^ and shearography among others^17,18^. Standard and well-known non-destructive testing methods using Lamb waves are used for large objects and high excitation frequencies—up to 6 MHz^1,19,20^. In our previous work, using holographic vibrometry^21,22^, we were able to investigate hidden defects in steel-polyamide laminates joined with a thin layer of epoxy adhesive and aluminium-polyamide laminates connected only adhesively (without any glue). We compared the amplitude and phase patterns on a sample excited to vibrations with frequencies in the range of 200–30 kHz. In both cases a debonding area could be detected and localized by measuring differences in vibration patterns on both sides of the sample.

In this study, we extended our method and undertook the observation of antisymmetric $A_0$ Lamb waves at different vibration frequencies of aluminium laminate samples with three different polymers and used their behaviour to investigate the presence of hidden defects^1,23^. Lamb wave propagation in an isotropic medium is well-defined for high frequencies (100 kHz and above). This is not the case for laminates and for a relatively low frequency range (up to 30 kHz in our case). We wanted to focus in our study on the effect of the defect on laminates in the low frequency range (including the audio range) for vibrations that occur in everyday life, e.g. in commonly used devices such as household appliances like washing machines^24^ or ultrasonic animal repellents^25^ and also in public places like railway stations or even libraries (due to i.a. public-address voice-alarm and transportation^25–28^).

We used digital holographic vibrometry (DHV) to determine the propagation of Lamb waves simultaneously on the metal and polymer side of the laminate. Observation of both sides is very useful especially for laminates. In this way, the quality of the connection of laminates made of blends of aluminium and polymers was assessed. Our method allows us to test samples with dimensions suitable for further strength tests. This is useful for routine component testing in a laboratory, as well as in an industrial production environment.

## Materials

In the case of joining materials with a large mismatch in the thermal expansion coefficients, like aluminium and polymers (see Table 1). Yamada et al. have observed that the Young’s modulus decreased when debonding appeared^29^.

**Figure 1:**
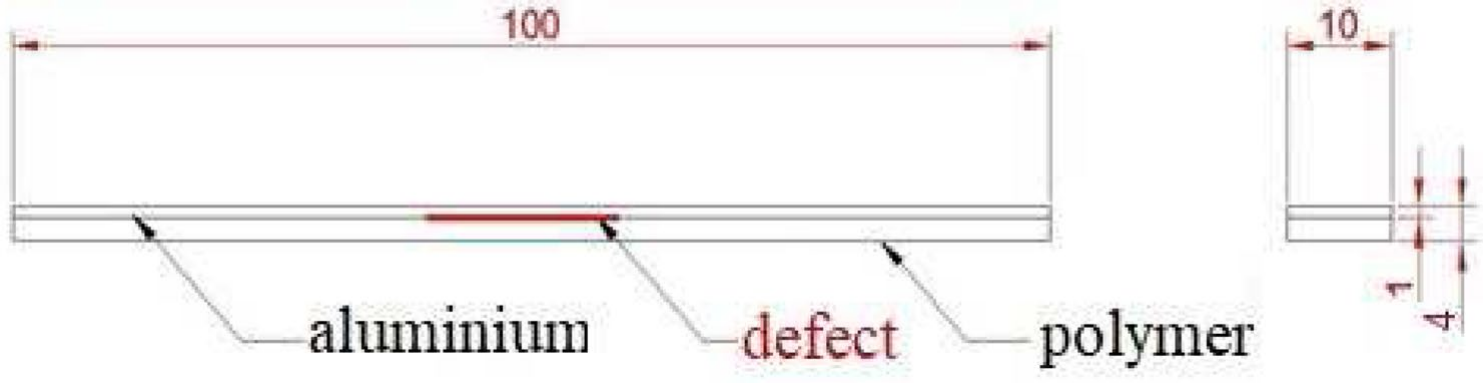
Sideview of a sample with a defect indicated; sizes are in mm.

We made three sets of polymer-metal laminates, connected only adhesively during compression moulding. Each set contained samples with and without defects. The defects varied in size: 10 to 15 mm broad across the full width of the sample (see Fig. 1).

Each set contains specimens with different polymers :

- Polylactide (PLA, *Ingeo Biopolymer 6400D*)—an eco-friendly, biodegradable, biocompatible, biopolymer used for packaging of short shelf life products and a promising material for biomedical applications^30^.
- Polyvinylidene fluoride (PVDF, *Arkema Kynar Flex 3312 C*)—excellent piezoelectric properties, mechanical strength and thermal stability, good processability and chemical resistance, applicable, e.g. in sensors and actuators, spin-valve devices, magneto-electric materials, energy harvesting applications and tissues^31^.
- Polycarbonate (PC, *Lotte Advanced Materials Infino S.C.- 1229UR*)—characterized by good processability, high impact resistance and safety, used e.g. in sport lenses and safety goggles^32^.

**Table 1:** Materials properties.

Mechanical properties	PLA	PVDF	PC	Al
Young’s modulus [GPa]	3.3	1	2.3	70.5
Coefficient of thermal expansion [$10\,^{-5}/\text {K}$]	4.1	12.8	7	2.37
Density [$\text {g}/\text {cm}\,^3$]	1.21	1.78	1.20	2.68

For all laminates, a 1 mm thick aluminium sheet PA11 (AW-5754) with dimensions of a single metal sheet 100 $\times$ 10 mm$^2$ (length $\times$ width) was used, a standard sample size for testing laminates.

Prior to the connection process, the polylactide and polycarbonate granulates were dried at 80 $\,^{\circ }\text {C}$ for 24 h, using a cabinet dryer.

The metal sheets were placed into the basket of an ultrasonic cleaner, submerged in acetone for a cycle time of 15 min, then rinsed with distilled water and cleaned with ethylene alcohol prior to the joining procedure to remove surface contamination.

The metal-polymer laminates were produced by compression moulding using a hydraulic press. We used a rectangular mould with cavity dimensions of 100 $\times$ 100 mm$^2$ and 4 mm height to prepare the samples.

Aluminium plates were placed in the cavity at 10 mm distance from each other. PTFE strips were placed directly on the metal plate to implement defects of metal-polymer bonding in the samples. The remaining space was filled with polymer granules. The metal and polymer inserts were kept in the closed mould for 10 min at temperature of :

- 200 $$\,^{\circ }\text {C}$$ for PLA,
- 230 $$\,^{\circ }\text {C}$$ for PVDF,
- 280 $$\,^{\circ }\text {C}$$ for PC.

Thereafter, a pressure of about 15 MPa was applied for 15 min, followed by cooling the laminate in the mould under pressure until full solidification. The PTFE strips were removed during post-processing.

## Methods

All samples were measured with a digital holographic vibrometer Optonor Vibromap 1000^33^ to examine the phase velocity of the Lamb waves. This set-up can register the vibrations of a pattern of 640 $\times$ 480 points simultaneously.

**Figure 2:**
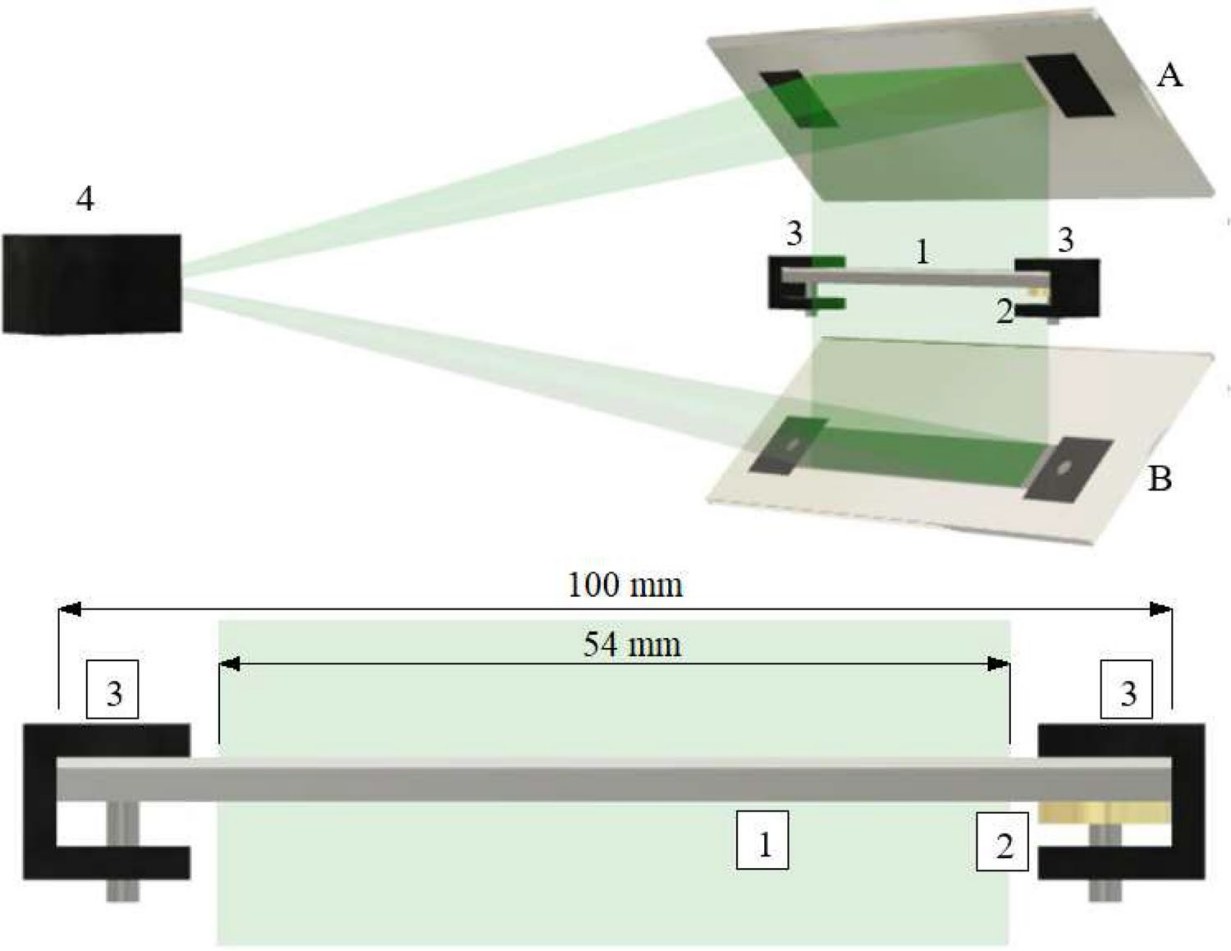
Experimental setup with the sample holder and the piezo transducer: 1: sample, 2: piezo transducer, 3: sample holders, 4: Vibromap, A: the mirror reflecting light from the upper surface of the sample, B: the mirror reflecting light from the lower surface of the sample reflected in the mirror^21^.

The set-up we designed allowed us to observe simultaneously an area of about 10 $\times$ 60 mm$^2$ of both upper and lower surface of the sample during a single measurement^21^ (see Fig. 2). The digital holographic vibrometer is basically a Michelson interferometer set-up with a Nd:YAG laser with a wavelength of 532 nm as light source. The reference arm is an optical fiber enclosed in the Vibromap and the measurement arm uses the sample as reflector. The average of several holograms is stored in a .mat file, containing the amplitude and the phase of the hologram. A detailed description of the set-up is given in Ref.^21^.

**Figure 3:**
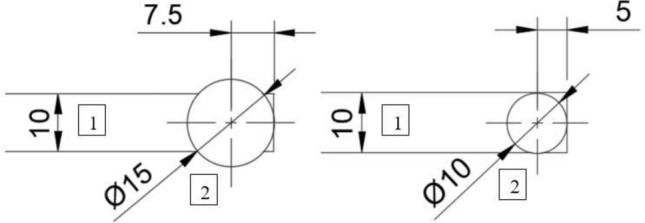
Botton view of a sample with position of both types of piezo trandsucers used: 1: sample, 2: piezo transducer; left: KingState KPE-827, right: PI P-010.00P; sizes are in mm.

A piezo transducer (PZT) was placed on the polymer side of the laminate in direct contact with the sample surface and clamped along with the sample—the laminate was clamped at both ends. We used two types of PZT (see Fig. 3): a PI P-010.00P (with a resonance frequency of 129 kHz) and a simple piezo audio transducer KingState KPE-827 working in a frequency range of 200 Hz–60 kHz (see Fig. 3). We compared data obtained for both types of transducer, and the results we got were consistent. The samples were continuously excited during measurement, to a harmonic vibration with an amplitude of a few to 50 nanometres. The frequency was varied from 1 to 30 kHz in steps of 100 Hz. This enabled us to observe antisymmetric $A_0$ Lamb waves—this type of wave is dominant below 100 kHz^34^. With the open source software Dispersion Calculator 2.0 provided by DLR (German Aerospace Center) for calculating dispersion curves we got for aluminium and polycarbonate results similar to our experiments^35^.

Special software, written by us in the programming language R^36^, was used to compare oscillations of both sides of the sample. The software extracts the amplitude and phase matrices from the raw data produced by the Vibromap software (version VibroMap_B4_nov15) of the vibrometer and visualises these data in different ways (see Figs. 9, 10). It also can filter the data to reduce noise, cut out relevant parts of the image and align the images of the opposite parts of the samples. Furthermore, the sign of the amplitude of the lower side is inverted. In this way, a positive amplitude indicates an upward movement on both sides. The vibromap defines a positive amplitude as towards the detector, in our set-up away from the centre plane of the sample. The data provided by DHV give us the amplitude of an array of vibrating points on the surface of a sample as well as the phase of the oscillation of each point at a certain (arbitrary) time of the vibration period. Using this information, we can reconstruct the behaviour of Lamb waves on the surface.

## Results and discussion

We tested, using DHV, aluminium and polymer only samples and three sets of laminates (see “Materials” section). In total, 30 samples were tested and each sample was tested several times, depending on the sample, but at least three times. We investigated the behaviour of the vibrating samples at low frequencies (up to 30 kHz) in terms of:

- the frequency range for which regular Lamb waves could be observed,
- the Lamb wave amplitudes,
- the Lamb wave propagation velocity depending on the frequency.

We used also amplitude and phase diagrams to localize bonding defects in the laminates.

### Lamb-wave frequency observation range

**Figure 4:**
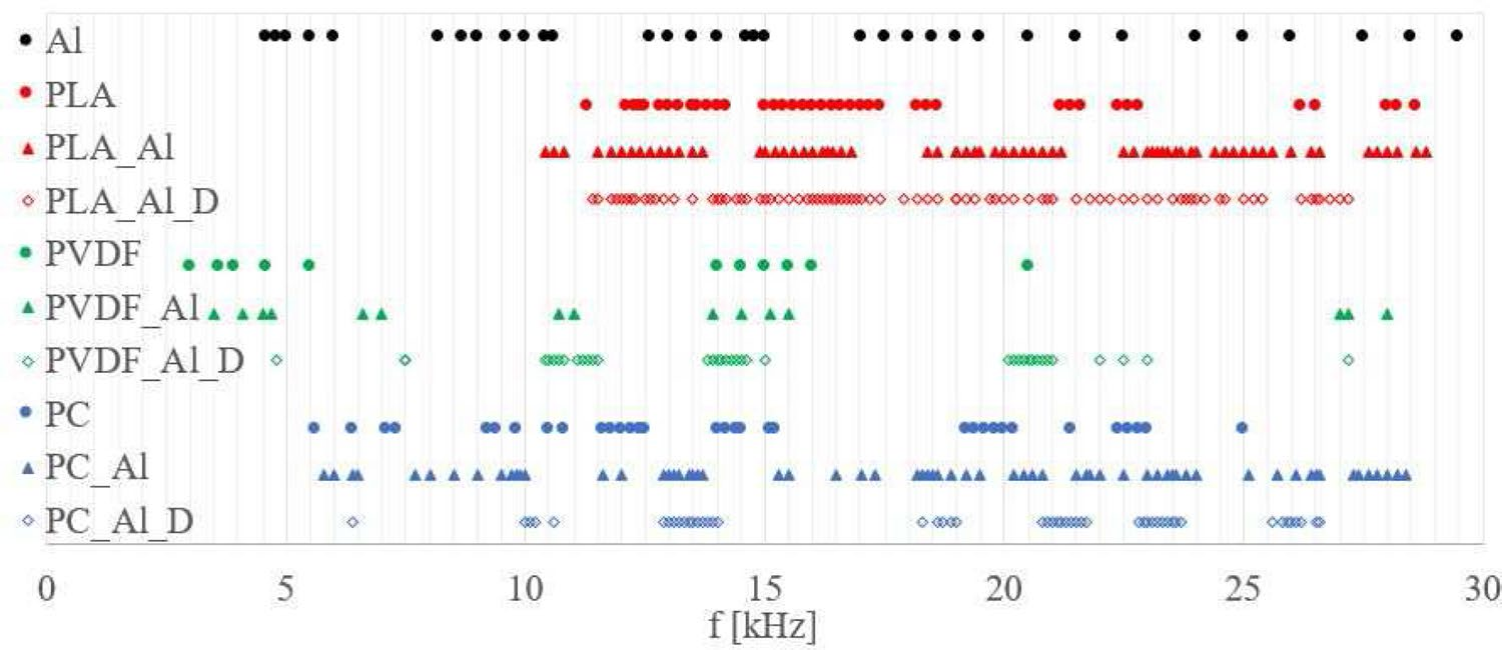
Frequencies for which Lamb-waves could be observed for aluminium and polymer only samples and laminates without and with a defect.

The whole frequency range between 200 Hz and 30 kHz was scanned for all types of samples (including a 1 mm thick aluminium only plate and a 3 mm thick polymer only sample. Width and length of these samples were the same as in the laminate samples. The *Lamb-wave Frequency Observation Range* (LFOR, see Fig. 4) is the frequency range for which Lamb waves were clearly visible (see Figs. 9 and 10). Samples with a specially made defect were coded with a D in the label (see the first column in Fig. 4 and in Figs. 6, 7, 8).

Aluminium only resp. polymer only were tested prior to laminate testing. For a pure aluminium plate we could observe Lamb-waves throughout the whole range from around 5 kHz up to 30 kHz. The polymer only samples had a different LFOR depending on the material, only covering about 20 kHz: 10–28 kHz for PLA, 3–22 kHz for PVDF and 5–25 kHz for PC (see Fig. 4).

For PVDF transmission of regular waves took place only for frequencies close to 5 kHz, 15 kHz or 20 kHz. For PLA and PC samples the gaps in the LFOR were much smaller.

In case of the polymer-aluminium laminates we noticed that the connection of pure polymer with aluminium led to a broadening of the LFOR. The gaps in the LFOR’s were also smaller. These effects were well visible for the PVDF_Al and PC_Al laminates. We observed that those samples transfer higher frequencies than pure PVDF or PC ones. For PLA_Al laminates however, the LFOR hardly changed compared to the LFOR of pure PLA samples, but started from higher frequencies (around 10 kHz). The presence of defects in the laminates diminished the LFOR compared with those of the fully bonded laminates. There was a slight tendency of LFOR’s to return to the ranges observed for the pure polymers.

Based on the amplitude of the Lamb waves, we found the following transmission properties of pure samples and laminates. The piezo transducer was placed on the polymer side as mentioned in “Methods” section and the excitation was always the same. As expected the best vibration transmission was shown by aluminium only samples—with the highest vibration amplitude of approx. 20 nm. For polymer only samples the vibration amplitudes were respectively 12 nm for PLA, 5 nm for PVDF and 15 nm for PC.

The Lamb-wave amplitudes for the laminates were different on both sides of the sample, especially for the PLA_Al and PVDF_Al laminates. Vibrations of the semi-crystalline PLA and PVDF polymer parts of the laminates were resp. about 10–20% and 40–50% weaker than for the metal part of the sample. The occurrence of a defect in the laminate increased the disproportion between metal and polymer vibrations, even up to 60–70% for PVDF_Al_D and up to 25% for PLA_Al_D. Only in the case of PC_Al laminates were the vibration amplitudes of the amorphous polymer part almost equal to those of the aluminium part (max. 5–10% different), even for laminates with a defect (PC_Al_D). For all types of laminates with a defect, there was however a phase shift between the vibrations of the metal and the polymer part.

### Lamb-wave velocity

**Figure 5:**
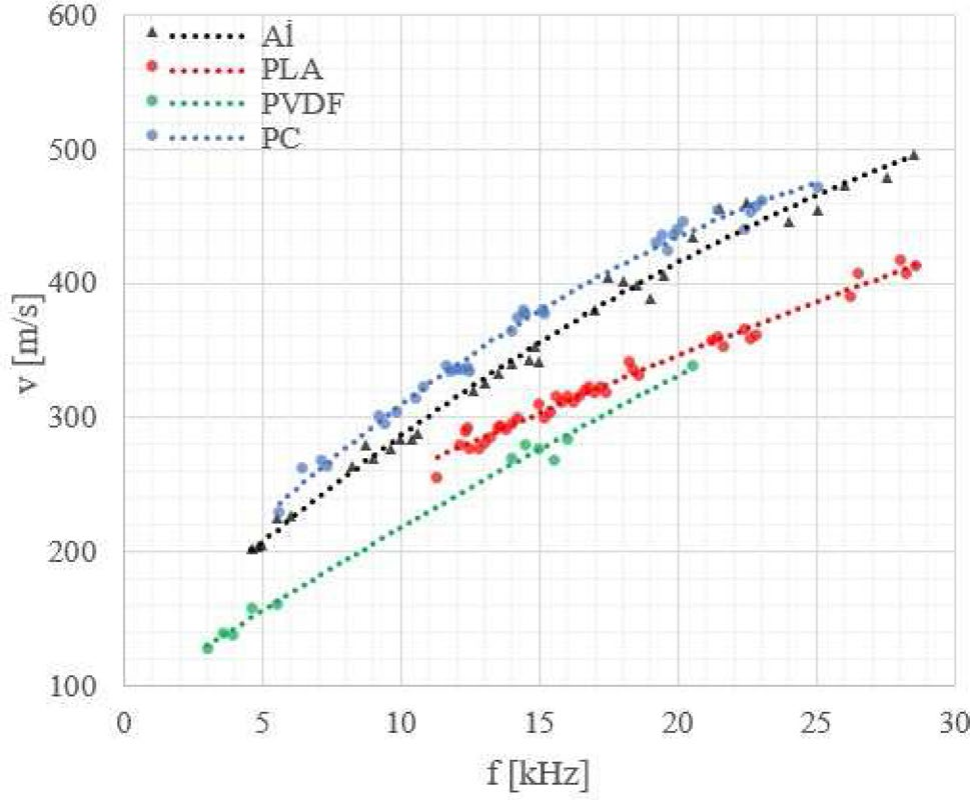
Comparison of the Lamb wave phase velocities for pure polymer and aluminium samples.

**Figure 6:**
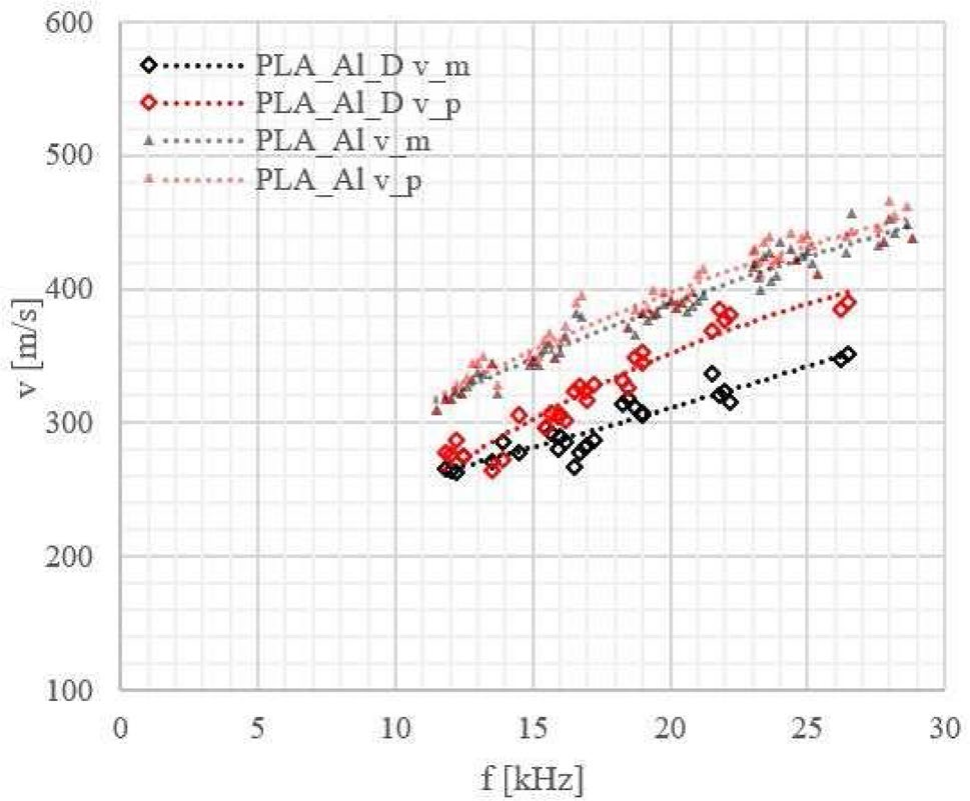
Lamb wave phase velocities for PLA_Al laminates without and with a defect:_D_; measurement errors: $\Delta$f = 1 Hz, $\Delta$v = 10 m/s. Velocities of the Lamb waves are marked: in black (v_m) for the metal part, in red (v_p) for the polymer part, $\triangle$ for laminates without and $\diamond$ for laminates with a defect.

**Figure 7:**
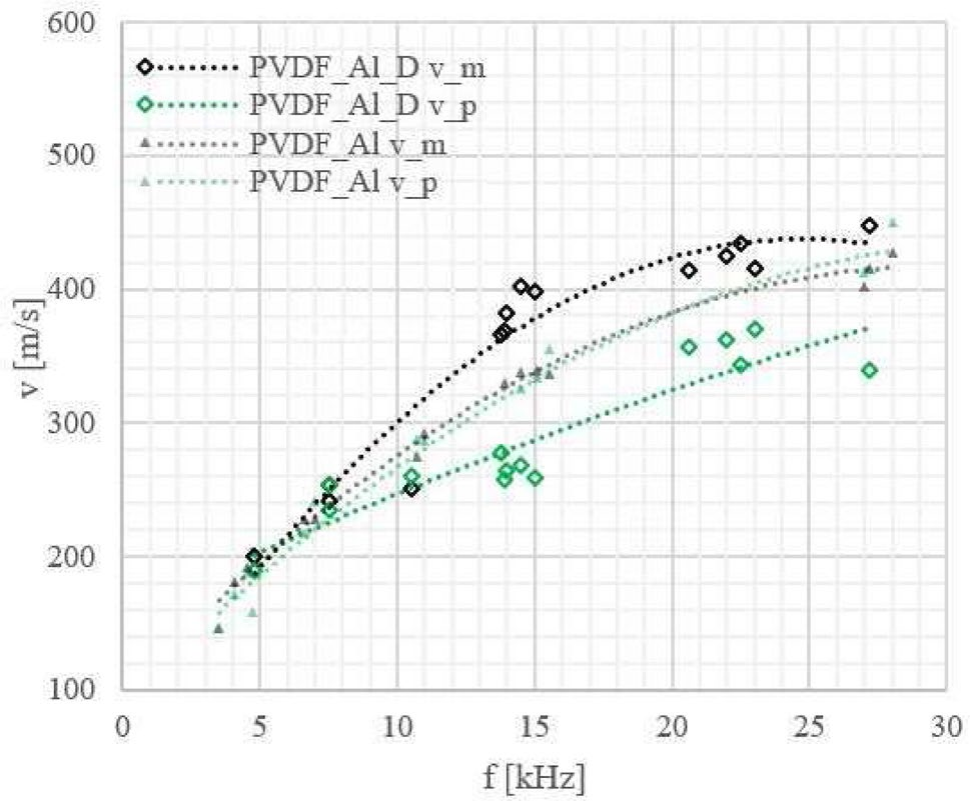
Lamb wave phase velocities for PVDF_Al laminates without and with a defect:_D_; measurement errors: $\Delta$f = 1 Hz, $\Delta$v = 10 m/s. Velocities of the Lamb waves are marked: in black (v_m) for the metal part, in green (v_p) for the polymer part, $\triangle$ for laminates without and $\diamond$ for laminates with a defect.

**Figure 8:**
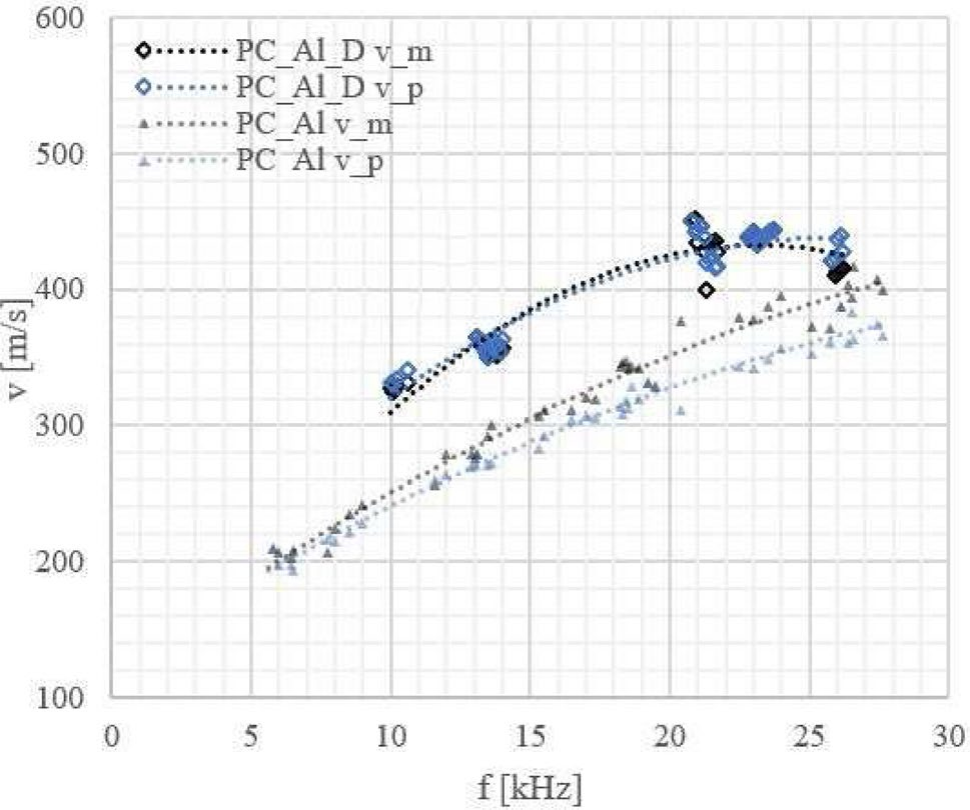
Lamb wave phase velocities for PC_Al laminates without and with a defect:_D_; measurement errors: $\Delta$f = 1 Hz, $\Delta$v = 10 m/s. Velocities of the Lamb waves are marked: in black (v_m) for the metal part, in blue (v_p) for the polymer part, $\triangle$ for laminates without and $\diamond$ for laminates with a defect.

**Figure 9:**
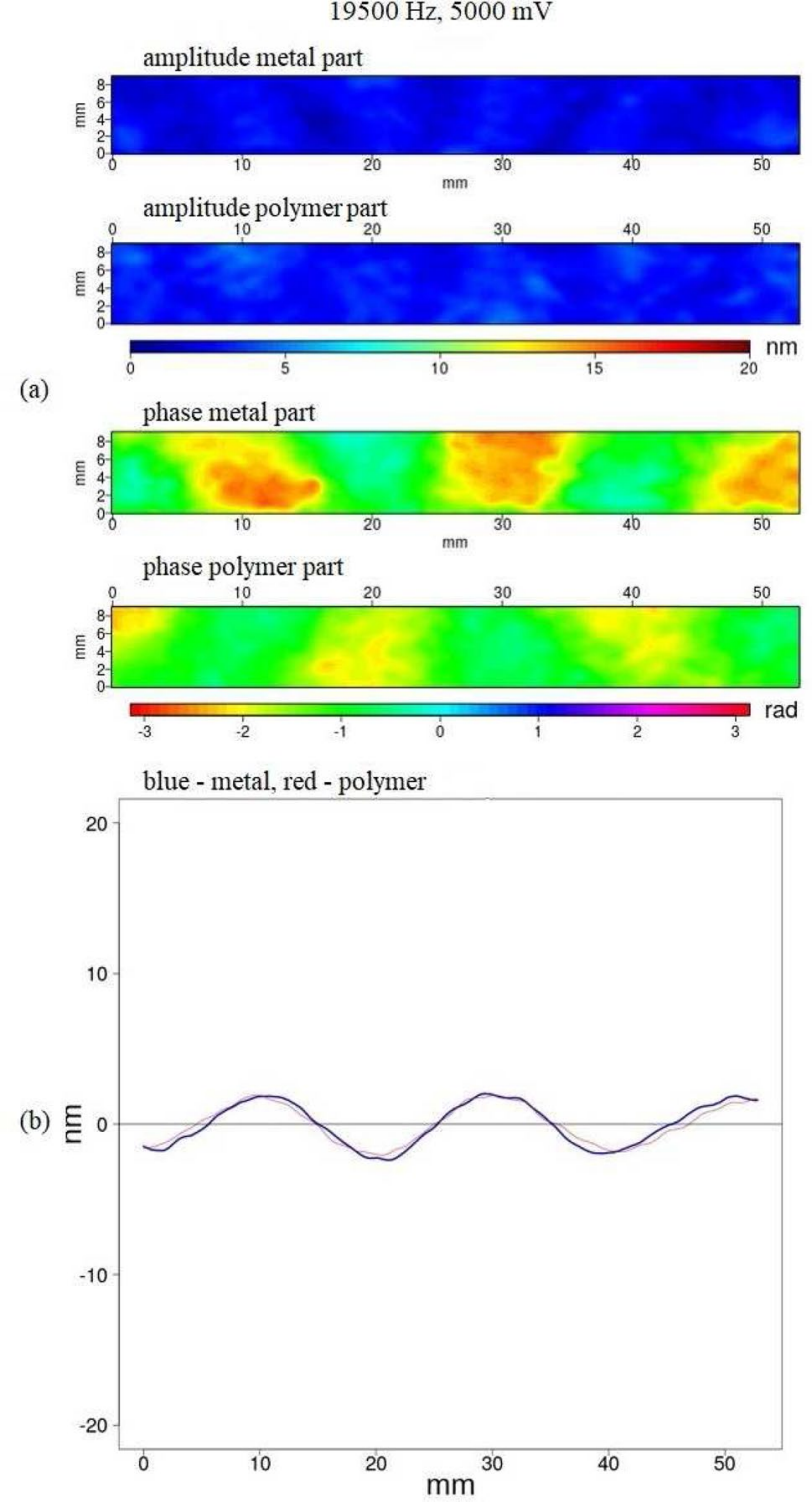
(**a**) Amplitude and phase pattern of a PLA_Al laminate sample without a defect. (**b**) The amplitude data from (**a**), averaged along the y-direction; blue—aluminium, red—polymer. The colour bars indicate the amplitude in nm, the phase in rad. The size of the visible part of the sample is 54 $\times$ 9.5 mm$^2$. The defect is located around 28 mm.

We also recorded velocity versus frequency curves of the Lamb wave phase velocity (see Figs. 5, 6, 7, 8) to check how a laminate defect affects these curves. The velocity of the anti-symmetric $A_0$ mode was determined using the 2D amplitude plots of the visible part of the specimen (approximately 54 mm) (see Fig. 9). From Fig. 9b, the amplitude averaged across the width of the sample, we could measure the Lamb wavelength and because we know the excitation frequency we can calculate the velocity.

**Figure 10:**
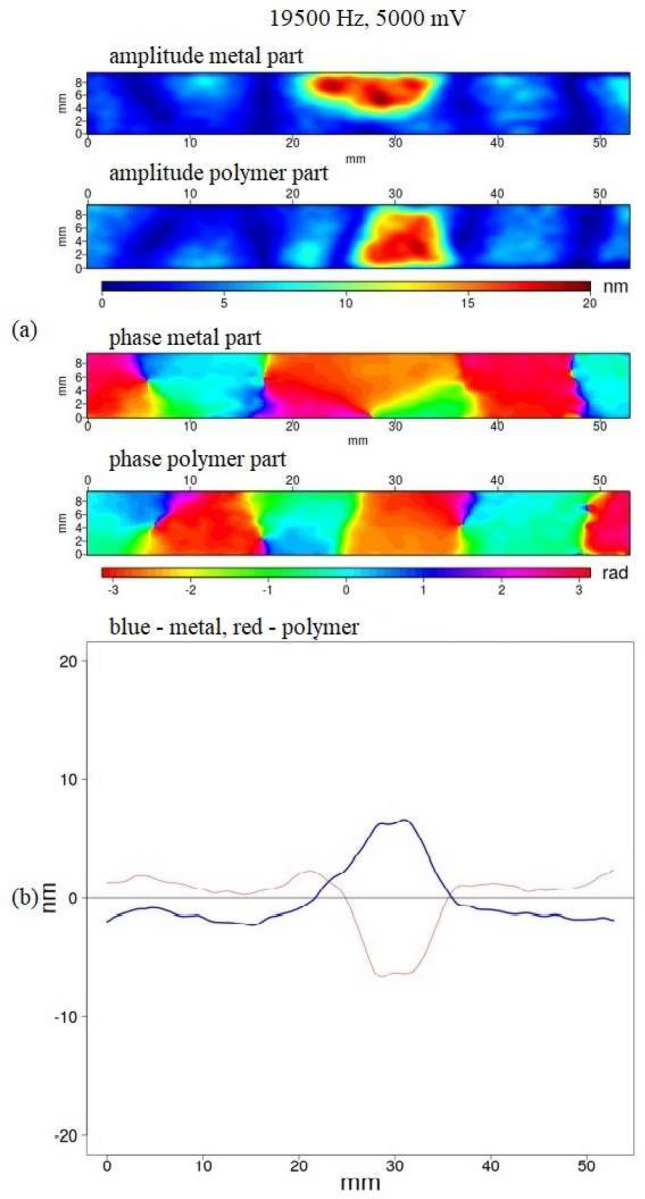
(**a**) Amplitude and phase pattern of a PLA_Al laminate sample with a bonding defect. (**b**) The amplitude data from (**a**), averaged along the y-direction; blue—aluminium, red—polymer. The colour bars indicate the amplitude in nm, the phase in rad. The size of the visible part of the sample is 54 $\times$ 9.5 mm$^2$.

We used a fixed amplitude range in our visualisation in Figs. 9 and 10 to easily compare the images for different frequencies. In some cases this made amplitude patterns less visible due to the small value of the amplitude. We could have used variable amplitude ranges, but also when the amplitude was small compared with the amplitude range the phase pattern indicated that we could use the amplitude data (see Fig. 9). Using a fixed amplitude range made it easier to compare different measurements.

Although it is not always possible to calculate the Lamb-wave velocity for the frequencies at which the defect is visible (see Fig. 10), we could observe that there is still a difference between the Lamb-wave velocity for samples with and without a defect throughout the wide frequency range (see Figs. 6, 7, 8).

Figure 5 shows the velocity versus frequency curves for pure polymer (3 mm thick) samples. Additionally a curve is provided for a pure aluminium sample (1 mm thick). As can be seen in Fig. 5, the wave velocities are in the frequency range up to 30 kHz lower in the samples with semi-crystalline, PLA and PVDF, than in the amorphous, PC, sample and in the metal one, which are closer to each other.

In Figs. 6, 7 and 8 the results for metal-polymer laminates without and with a bonding defect are presented.

Change in the behaviour of the Lamb waves was observed due to the coupling of waves propagating in metal and polymer parts enabled by the adhesion connection between them. For samples without a defect Lamb wave are coupled strongly, contrary to the Lamb waves in the samples with a defect.

For aluminium-polymer laminates without bonding defects we observed that the velocity of the Lamb waves is mainly determined by the metal part of the sample and only slightly dependent on the type of polymer used.

For laminates with a specially made defect the Lamb wave velocities in metal and polymer start in the case of PLA_Al_D and PVDF_Al_D laminates to differ significantly from each other (see Figs. 6, 7). The largest differences between the velocities in the metal and polymer layer are observed for PVDF_Al_D. This is consistent with the results we obtained from the comparison of vibration amplitudes.

In the case of PC_Al_D laminates, we could not observe differences in the Lamb wave phase velocity for the polymer and metal parts. The differences in the behaviour of the laminates without and with a defect were however clearly visible (see Fig. 8). The Lamb-wave phase velocities for a given frequency were higher in case of a laminate with a defect in comparison to one without a defect. We observed for all laminates with a defect that the Lamb wave velocity in the polymer part was becoming similar to the velocity in pure polymer (see Fig. 5).

From a practical point of view, velocity measurements in both parts of a metal-polymer laminate sample at frequencies close to 20 kHz can be a sufficient guide for detection of defects 10–15 mm wide.

### Defect localization

In order to detect and localize a defect the amplitude and phase patterns of laminate vibrations were studied. For samples without a defect vibration of the metal and polymer parts of the sample are compliant, both sides of the sample move together and in the same direction. An antisymmetric Lamb wave A$_0$^19,20,23^ can be observed (an example is shown in Fig. 9b). The Lamb wave can be represented in amplitude as well as phase pattern.

We were able to detect and localize defects for all laminates measured by DHV. For laminates with a defect the Lamb wave loses its regularity; in some cases torsional vibration in the area of the defect could be observed (see Fig. 10). The occurrence of a defect can additionally increase the amplitude differences between the metal and polymer vibrations.

The LFOR for which we could observe defects in laminates varies more depending on the defect size than on the polymer material used. In general, the smaller the defect the higher the excitation frequency must be in order to enable observation: defects of about 15 mm could be observed around 13–16 kHz and smaller ones (about 10 mm wide) could be detected around 19–22 kHz.

## Conclusions

We used two semi-crystalline polymers (PLA and PVDF) and one amorphous polymer (PC) as components of the laminates.

Amplitude and phase patterns made it possible to locate a defect. The LFOR for which a laminate defect could be observed depends on defect size rather than on the polymer material used.

For all aluminium-polymer laminates without a defect, the Lamb-wave propagation in the polymer follows the one observed in the metal. This indicates good bonding of both layers and shows that all polymers tested can be successfully used to produce a stable laminate. Because we observe simultaneously the metal and the polymer side of the sample, our set-up requires access to both sides.

The presence of a bonding defect manifests itself in several ways. In all cases the defect limits the LFOR. It also significantly changes the anti-symmetric $A_0$ Lamb wave velocity versus frequency curves. The discrepancies in the curves for the metal and the semi-crystalline polymers becomes visible at frequencies above 10 kHz. At a frequency of 15 kHz the Lamb wave velocity in the polymer part of the PVDF_Al_D laminate with a defect was even about 40% lower than in the aluminium layer.

The presence of a defect in a laminate increased the difference between the metal and the polymer vibration amplitudes in the case of semi-crystalline polymers. Especially for PVDF, which shows the weakest vibration transmission among the materials tested, this difference can increase from 40–50% (for PVDF_Al) to 60–70% for PVDF_Al_D. For amorphous PC_Al however no big difference was observed in the vibration amplitudes, even for laminates with a defect. Hence, the amplitude criterion may not be effective in the case of laminates with polymers with good vibration transmission in the test range of low frequencies.

It should be stressed that the NDT method presented requires only a limited time to test the quality of the bonding between the two materials of a laminate. Using DHV to detect Lamb waves is a useful extension of the standard laminate tests that also can be carried out on a production line.

## Data Availability

The datasets used and/or analysed during the current study are available from the corresponding author on reasonable request.
